# Origin and Role of DNA Damage in Varicocele

**Published:** 2012-12-17

**Authors:** Mohammad Hossein Nasr Esfahani, Marziyeh Tavalaee

**Affiliations:** 1 Department of Reproduction and Development, Reproductive Biomedicine Research Center, Royan Institute for Biotechnology, ACECR, Isfahan, Iran; 2 Department of Embryology, Reproductive Biomedicine Research Center, Royan Institute for Reproductive Biomedicine, ACECR, Tehran, Iran; 3 Isfahan Fertility and Infertility Center, Isfahan, Iran

**Keywords:** Varicocele, DNA Damage, Repair

## Abstract

Varicocele is considered as one of the main etiologies of male infertility. Along with altered semen parameters, increased DNA fragmentation is believed to play an important role in varicocele-induced infertility. DNA damage may result from intra- or extra-testicular factors. Among these, apoptosis, abnormal chromatin packaging and oxidative stress are the most researched and are addressed in this review. Significant evidence suggests that varicoceles have a harmful effect on testicular function and a varicocelectomy not only prevents progressive decline in testicular function, but also reverses the damage. However, the degree to which varicocele repair improves pregnancy rates and the success of assisted reproductive technology (ART) remains controversial. Therefore, the role of varicocele repair on DNA fragmentation is also discussed.

## Introduction

Abnormally dilated veins in the pampiniform plexus define varicocele, which is historically associated with male factor infertility (1). Even though varicoceles have been detected in boys as young as ten years of age, it is commonly believed that this condition may begin with the onset of puberty, at around the age of 15 (2, 3). The incidence of varicocele in adolescents is approximately 15% and, in the general population, it ranges from 4.4 to 22.6% (4). What distinguishes varicocele is its higher incidence in the infertile population; 21-41% in men with primary infertility and 75-81% in those with secondary infertility (5, 6). In addition, it causes a decrease in both semen quality and testicular volume.

The effects of varicocele are long-term and progressive, therefore the fertility potential of individuals afflicted with varicocele declines by the time they desire to achieve fatherhood (7). However, what has made the role of varicocele in fertility enigmatic is the fact that fertile men with varicocele are able to be father while infertile men with varicocele have lower chances for fertility. Surgical correction of varicocele improves their fertility potential or assisted reproductive technology (ART) outcome post-varicocelectomy (8). A likely explanation is the heterogeneity observed in the varicocele population, which includes different grades of varicocele, clinical diversity, location (bilateral vs. unilateral), duration of varicocele, individual genetic background, and social, economic, and geographical status of these individuals (6, 9, 10). Moreover, the pathophysiology of varicocele remains unclear.

Despite the availability of different procedures to diagnose varicocele, physical examinations and scrotal ultrasounds remain the most commonly used methods. Varicocele is graded at the time of the initial physical examination according to the Dubin grading system (I-III), where grade II is palpable without the Valsalva maneuver and grade I is not visualized, rather it is only palpable by the Valsalva maneuver (11- 13). The term clinical varicocele refers to varicoceles detectable by palpation or visual inspection.

### Treatment

Considering the enigma regarding varicocele, its treatment is also controversial. There are two classes of thought regarding the treatment of varicocele. One group believes that its treatment improves semen parameters and fertility whereas the other believes there is no significant improvement post-varicocelectomy (6, 14, 15).

What is important in these studies are the control groups and sample sizes. Some studies have shown that the percentage of pregnancies in varicocele individuals after varicocelectomy was not statistically higher in comparison to those who underwent no treatment (14, 16). However, the current guidelines or the general consensus among the clinicians is that varicocele individuals with decreased testicular size and abnormal seminal parameters must be treated (17, 18). A recent meta-analysis suggests that the spontaneous pregnancy rate increases following repair of the varicocele. However, if confiding variables or heterogeneity between the clinical trials are taken into consideration, this effect would become insignificant or less clear (6).

In addition, the rate of spontaneous abortion in the spouses of post-varicocelectomy responders has been reported at approximately15% and remains an issue of concern (19, 20). Increased levels of sperm DNA fragmentation have been proposed as a possible cause for low-quality embryos and spontaneous abortion. In this meta-analysis, the authors have shown that varicocele repair significantly improved sperm concentration and motility, yet they were unable to reach a conclusion on sperm morphology due to variations between different studies (6). These controversies suggest that more robust parameters with threshold should be defined to detect individuals who may benefit from varicocelectomy. To achieve this, different authors have assessed sperm functional parameters, such as DNA fragmentation, which will be discussed in the course of this review.

### DNA damage and varicocele

Spermatogenesis is a complex process by which the male germ cell proliferates and matures through meiosis from diploid spermatogonia to haploid spermatozoa. Although a small percentage of spermatozoa from fertile men possesses detectable levels of DNA damage, which can occur at any step of spermatogenesis (21, 22), a higher degree of DNA fragmentation is clearly associated with male infertility. Many factors, including intra- or extratesticular factors, may be involved in this process (21, 23). Abnormal chromatin packaging, abortive apoptosis, and extreme production of reactive oxygen species (ROS) are factors which may lead to DNA damage (24-26). In addition, extra-testicular factors such as age, drugs, cigarette smoking, genital tract inflammation, hormonal factors, varicocele, and testicular hyperthermia are among the main reasons for DNA damage (24).

A variety of methods have been used to evaluate the integrity of sperm chromatin and DNA. For evaluation of major proteins associated with DNA, researchers commonly use chromomycin A3 (CMA3) and aniline blue staining. Deoxynucleotidyl transferase-mediated dUTP nick-end labeling (TUNEL), the comet assay, sperm chromatin dispersion (SCD), acridine orange (AO), and sperm chromatin structure assay (SCSA) are used for DNA breaks. Recently our group presented a review article that discussed the methodologies, advantages, and disadvantages of these tests that was published in the International Journal of Fertility and Sterility (IJFS) (25). The current review focuses on the relation between internal testicular factors involved in DNA damage and their relation with varicocele.

### Apoptosis and varicocele

Germ cell death occurs during normal spermatogenesis in mammals and it is estimated to be responsible for the loss of up to 75% of potential spermatozoa. Spermatogonia are eliminated by apoptosis and only 25% of the theoretically expected number of primary spermatocytes are produced from the original population of spermatogonia (27, 28). In addition, 20% of spermatocytes and spermatids are frequently removed by selective apoptosis (29). Therefore, the presentation of apoptotic markers such as phosphatidylserine externalization, DNA fragmentation, and Fas expression are considered a part of this process (30, 31). Increased apoptosis, necrosis, and degeneration of sperm in individuals with varicocele may suggest that a redundancy of spermatogenic cells by apoptosis is facilitated in these patients. At least three pathways have been proposed: i. excess heat, ii. androgen deprivation at the testicular level, and iii. accumulation of toxic agents, including the products of cigarette smoke, such as cadmium (10).

A higher population of sperm with fragile DNA has been observed upon heat treatment. According to research, heat treatment is associated with poor capacitation characteristics and apoptosis (32). Therefore, it is plausible to expect that exposure to poorly regulated genital temperatures may cause spermatozoa to undergo significant apoptosis or necrosis during spermatogenesis in varicocele individuals, and at its extreme it may lead to hypospermatogenesis and spermatogenetic arrest. At the spermatid level, a predominant phenotype has been reported by Lin et al. in varicocele individuals. This is the reason for improvement in semen parameters in azoospermic men with maturation arrest post-varicocelectomy (33, 34).

It has been shown that Sertoli cells can begin and regulate germ cell apoptosis via the apoptosis-stimulating Fas system, as characterized by the interaction between Fas and its ligand (30 , 35). Del Giudice et al. evaluated FasL mRNA levels using reverse transcription and real-time quantitative polymerase chain reaction (RQ-PCR) in adolescents with and without varicocele. They concluded that FasL mRNA levels were higher in varicocele individuals compared to those without varicocele. In addition, they observed a significant negative correlation between sperm concentration and FasL mRNA levels. These findings suggested that individuals with low sperm concentrations have high FasL mRNA levels (36 ).

Wu et al. also evaluated apoptosis markers in varicocele individuals. Their findings showed increased externalization of phosphatidylserine, mitochondrial dysfunction, and nuclear DNA damage in fertile and varicocele individuals. This has suggested that an association may exist between certain apoptotic mechanisms and varicocele, which possibly originates in the mitochondria of spermatocytes, resulting in DNA anomalies or fragmentation in the nucleus of these cells (37 ).

### Protamine deficiency and varicocele

Spermatogenesis is considered a continual process. However, in order to make this process more comprehensible, it has been divided into three components: spermatocytogensis, reduction division, and spermiogenesis (38 ). During normal spermiogenesis, 85% of histones are replaced with protamines, which results in sperm chromatin condensation (39 ). According to Oliva, chromatin condensations are essential for: i. protecting paternal genes from nucleases, mutagens, toxins, and heavy metals in the testis or spermatozoa, ii. the generation of a hydrodynamic nucleus to facilitate genome transportation and fertilization, and iii. the removal of proteins and transcription factors from the spermatid, which can lead to the formation of a blank paternal genetic message free of epigenetic information and ready for reprogramming by the oocyte (40 ). Different authors have shown that varicocele adversely affects these processes. For reason El-Segini et al. evaluated chromatin condensation by aniline blue in infertile patients with varicocele and showed that they have significantly more impaired chromatin condensation than either the fertile group or those fertile individuals with varicocele (41 ). Thus, they concluded that one of the major spermiogenic insults affecting fertility in varicocele patients was a defect in the histone/ protamine exchange (40 , 41 ).

This defect has also been verified by other authors using different approaches, such as assessing protamine deficiency by CMA3 or showing the presence of excessive histone by anilineblue or toluidine blue (42 , 43 ). Our studies in varicocele individuals also confirmed defects in histone/protamine exchange and have further shown that this insult is rectified by varicocele repair (8 , 44 ). However, by assessing protamine deficiency in pregnant and non-pregnant partners of individuals who underwent varicocelectomies, our results have indicated no significant improvement in one group over the other. We can thus conclude that the defect in his tone/protamine exchange may be one of the mechanisms associated with varicocele, it may not be the major factor in the pathogenesis of varicocelectomy (8 ).

Hyperthermia is believed to play a key role in the pathophysiology of varicocele and the reduction of fertility rates (45 ). One of the responses of eukaryotic organisms to hyperthermia is production of heatshock proteins (HSPs) (46 ). HSPA2, a member of the 70-kDa family, is testis tissue-specific and has been shown to function as a chaperone protein that aids in protein folding (47 ). The deficiency of this gene in knockout mice or a mutation in this gene results in the arrest of primary spermatocytes at stage I of meiosis, leading to azoospermic or infertility in mice. This result shows an important role for HSPA2 in spermatogenesis (48 ). HSPA2 has been evaluated as a sperm maturity and function index (49 ). Our study showed that chronic hyperthermia induced by varicocele not only resulted in impaired semen parameters but also reduced HSPA2 expression when compared to the fertile population. Removal of this stress by varicocelectomy has been shown to improve semen parameters and increase HSPA2 expression (50 ). These improvements are likely achieved by the proper folding proteins involved in spermatogenesis including protamine, which is involved in DNA packaging and protection of DNA from damage.

### Reactive oxygen species (ROS) and varicocele

ROS are free radicals produced in biological systems that have an important role in capacitation, hyper-activation, and sperm oocyte fusion (51 ). In the presence of polyunsaturated fatty acids in the plasma membrane of sperm, ROS triggers a chain of chemical reactions called lipid peroxidation (51 ). It has been shown that ROS can damage DNA by causing deletions, mutations, and other lethal genetic effects (52 ). Moein et al. compared ROS levels in the seminal fluid of infertile men with varicocele and idiopathic infertility to fertile donors and showed ROS levels to be significantly higher in the former group. In addition, it has been shown that elevated scrotal temperature due to impaired circulation results in the accumulation of toxic metabolites, which is believed to be the source for ROS production in these individuals (53 ).

Apart from xenobiotics and the accumulation of toxic metabolites, the other key sources of ROS in semen are leukocytes and immature spermatozoa (sperm with residual cytoplasm) (54 ). Thus DNA damage in sperm may have been induced by ROS sources. Kemal Duru et al. have shown that exposing spermatozoa to artificially produced ROS significantly increases DNA damage (55 ). However, the effect of ROS on DNA fragmentation should only be considered in light of cellular antioxidant mechanisms and the antioxidants present in the secretom. In association with increased ROS and DNA fragmentation,a decrease in the level of antioxidants (including superoxide dismutase, glutathione peroxidase, catalase, and ascorbic acid) and total antioxidant capacity has been reported both in infertile and varicocele individuals (56 ). Some authors have reported a clear association between ROS levels and antioxidant capacity to sperm parameters in infertile and varicocele individuals (57 , 58 ). In addition, they believe that these aberrant levels of ROS and antioxidants are involved in the occurrence of oligospermia, sperm motility defects, and/or abnormal sperm morphology (59 ). Simsek et al. have suggested that this effect is mediated via cellular apoptosis (60 ). A meta-analysis by Agarwal et al. (5 ) has shown significantly higher ROS and lower total antioxidant capacity levels in the varicocele population compared to fertile men, which may have resulted from abortive apoptosis and improper protamination. Chen et al. in a prospective study, have documented a reduction in ROS level following varicocele repair. Thus, varicocele repair may restore spermatogenesis, and improve semen parameters and decreases DNA damage via decreases in ROS levels (52 ).

### DNA damage and varicocele repair

Evaluation of DNA damage has been proposed as extra information regarding sperm quality and is a forecaster of fertility potential. In the previous section, various factors involved in DNA damage have been discussed. The management of infertile men with increased sperm DNA damage remains high, particularly in varicocele individuals. Some studies have reported that sperm DNA fragmentation to be significantly higher in varicocele individuals compared to those with normal semen parameters (50 , 61 ). Furthermore, DNA damage is associated with fertilization rate, spontaneous pregnancy, and pregnancy outcome post ART (62 , 63 ). It is important to confirm whether varicocele repair may resolve varicocele-induced DNA damage or factors involved in sperm DNA damage.

Many studies suggest that varicocele repair should be performed in infertile men who have clinical varicocele and abnormal semen analyses (8 , 64 ). Despite this consensus, the question of whether varicocele should be repaired in individuals with high DNA damage remains controversial. In a prospective trial, Smit et al. have stated that the percentage of DNA index by SCSA was reduced three months post-varicocele repair (14 ). In addition, Azadi et al. (44 ) and Zini et al. (65 ) showed the beneficial effect of varicocele repair on human sperm DNA damage. Previous studies have found no significant reduction in DNA damage three months post-varicocele repair. They propose that the duration of spermatogenic cycle is about 64 days, thus the reduction of DNA fragmentation should be assessed six months after varicocele repair.

Considering this overview of the research papers, we suggest that varicocele repair should be performed in varicocele individuals who have high DNA fragmentation in their semen samples prior to surgery.

## Conclusion

According to the literatures, this review suggested that varicocele has a detrimental effect on testis function and mainly spermatogenesis. Therefore, varicocelectomy could improve testicular function and sperm production.
